# Peripheral Nerve Block with Ropivacaine Ameliorates Acute Compartment Syndrome by Promoting Macrophage M2 Polarization via JAK-STAT Signaling

**DOI:** 10.3390/biomedicines14092065

**Published:** 2026-09-15

**Authors:** Yingying Deng, Yuan Pan, Tao Wang, Chaoran Hu, Kunzhi Zhu, Chao Xie, Chao Feng

**Affiliations:** 1 Clinical Medical College, Guizhou Medical University, Guiyang 550031, China; 17731186854@163.com (Y.D.);; 2 Department of Lower Extremity Trauma Orthopedics, Beijing Jishuitan Hospital Guizhou Hospital, Baiyun District, Guiyang 550005, China; 3 Department of Orthopaedics, Center for Musculoskeletal Research, University of Rochester Medical Center, Rochester, NY 14642, USA

**Keywords:** acute compartment syndrome, peripheral nerve block, ropivacaine, macrophage polarization, M2 macrophage, JAK-STAT

## Abstract

**Background:** Acute compartment syndrome (ACS) is a critical orthopedic emergency where increased pressure in a limb compartment leads to muscle ischemia, necrosis, and lasting dysfunction. While fasciotomy is the standard treatment, it often results in complications like infection and nerve damage, underscoring the need for effective non-surgical therapies to manage the inflammation causing tissue damage. Peripheral nerve block (PNB) with ropivacaine is clinically used for analgesia, but whether it can serve as a disease-modifying adjunct intervention for ACS beyond pain control remains unexplored. **Objective:** This study examined the potential of PNB utilizing ropivacaine to enhance outcomes in ACS and explored the underlying mechanisms related to macrophage polarization. We aimed to distinguish two unresolved questions: (1) whether ropivacaine-based PNB confers tissue-protective effects in ACS independent of analgesia; (2) what signaling pathway mediates ropivacaine-driven macrophage phenotypic switch under ACS-relevant inflammatory conditions. **Methods:** Using rats, an ACS model was established, with the animals being randomly allocated to five different groups: Control, ACS, sciatic PNB, femoral PNB, and combined PNB. The study assessed functional outcomes, histopathological changes, levels of inflammatory markers (interleukin-6 (IL-6), C-reactive protein (CRP), and markers of macrophage polarization (CD86, CD206). In parallel, RAW264.7 macrophages stimulated with LPS and IFN-γ were treated with ropivacaine (1–10 μg/mL), and M1/M2 markers, pro-inflammatory cytokines (TNF-α, IL-6, IL-1β), migratory capacity, and cytotoxicity were assessed. Differentially expressed genes and enriched pathways were identified through RNA sequencing, followed by mechanistic validation of JAK-STAT signaling using Western blotting, immunofluorescence, immunohistochemistry, and rescue experiments with the JAK2 inhibitor AG490. **Results:** PNB significantly improved all functional and histological parameters in ACS rats, with combined blockade demonstrating the greatest efficacy. Ropivacaine at 5 μg/mL promoted M2 macrophage polarization, evidenced by upregulated CD206 and arginase-1 alongside downregulated CD86 and iNOS, while reducing pro-inflammatory cytokine production without cytotoxicity. Ropivacaine also suppressed macrophage migration. Among 83 differentially expressed genes, RNA sequencing pinpointed JAK-STAT as the pathway with the highest level of enrichment. Mechanistically, ropivacaine activated JAK2-STAT3, reflected by increased p-JAK2/JAK2 and p-STAT3/STAT3 ratios. AG490 completely reversed ropivacaine-induced JAK-STAT activation, M2 polarization, cytokine suppression, and migratory inhibition in vitro, and similarly abrogated PNB-mediated functional recovery and histological protection in vivo. **Conclusions:** In vitro, ropivacaine directly promoted macrophage M2 polarization via JAK-STAT activation. In vivo, ropivacaine-based PNB ameliorates ACS, which may involve both direct immunomodulatory effects of ropivacaine and indirect biological consequences of peripheral neural blockade. This study provides the first pre-clinical proof-of-concept that ropivacaine PNB can act as an adjunctive disease-modifying strategy for ACS (beyond analgesia), and identifies macrophage JAK2-STAT3 as the critical molecular cascade responsible for this immunomodulatory effect. This identifies a readily translatable adjunctive strategy for ACS and establishes JAK-STAT as a potential pharmacological target for compartment syndrome-associated inflammation.

## 1. Introduction

Acute compartment syndrome (ACS) is a critical surgical emergency marked by increased pressure in a confined osseofascial space, leading to reduced blood flow to tissues, progressive ischemia, and irreversible neuromuscular damage [1,2]. Surgical fasciotomy has been the gold standard treatment for established ACS, aimed at decompressing the affected compartment and restoring tissue perfusion [1,2]. However, delayed intervention substantially increases the risk of irreversible muscle necrosis, neurologic deficits, and potential amputation [3]. In addition to the immediate surgical intervention, fasciotomy is associated with notable complications, such as substantial open wounds necessitating extensive soft tissue reconstruction, wound infection, delayed healing, prominent scarring, and chronic venous insufficiency. Muscle weakness, sensory deficits, chronic pain, and functional limitations are potential long-term outcomes that can greatly affect a patient’s quality of life [1,3]. The significant treatment-related complications highlight the urgent need for effective additional or alternative therapies to minimize tissue damage, reduce inflammation, and enhance recovery in ACS. ACS involves not only mechanical compression but also complex inflammatory and immune mechanisms. Increased intracompartmental pressure leads to “low-flow” ischemia, causing tissue hypoxia, which generates reactive oxygen species (ROS) and releases damage-associated molecular patterns (DAMPs) from dying cells [4]. The molecular signals activate immune cells present in the area and draw in circulating leukocytes to the site of injury, beginning an acute inflammatory process [5]. Significantly reducing microvascular dysfunction and muscle injury in experimental compartment syndrome models has been demonstrated by neutralizing these pro-inflammatory cytokines, highlighting inflammation as a potential therapeutic target [6].

In relation to ACS, macrophages have a key and diverse function in the inflammatory response to skeletal muscle injury [7]. In the event of tissue damage, inflammatory monocytes in the blood swiftly migrate to the injury site, where they become pro-inflammatory M1 macrophages in response to interferon-gamma (IFN-γ) and microbial agents, facilitating their ability to phagocytize cellular debris and secrete inflammatory mediators such as TNF-α, IL-1β, and monocyte chemoattractant protein-1 (MCP-1) [8]. The initial inflammatory phase is essential for the removal of necrotic tissue and the activation of muscle stem cells. Nonetheless, sustained M1 macrophage dominance prolongs tissue damage and hinders the regeneration process [7,8]. During healthy wound healing, M1 macrophages transition to anti-inflammatory M2 macrophages, marked by CD206, arginase-1 (Arg1), and interleukin-10 (IL-10), which aid tissue repair, reduce inflammation, and support muscle differentiation [7,9]. For skeletal muscle to regenerate effectively, the M1-to-M2 transition must occur in a specific order, and any imbalance in macrophage polarization can lead to pathological healing, fibrosis, and chronic inflammation [8,10]. The M2 macrophage phenotype comprises various subtypes, notably including M2a macrophages, which are induced by interleukin-4 (IL-4) or interleukin-13 (IL-13) and are characterized by elevated expression of transforming growth factor-beta (TGF-β) and arginase. Additionally, M2c macrophages, activated by interleukin-10 (IL-10), play a crucial role in promoting extracellular matrix remodeling and the resolution of inflammation [11,12]. Disrupting the polarization sequence, with prolonged M1 dominance or delayed M2 transition, leads to chronic inflammation, excessive fibrosis, and impaired recovery [7,10].

Peripheral nerve block (PNB) techniques are important for multimodal analgesia in lower limb surgeries, offering effective pain relief and minimizing opioid use and its side effects. Combined femoral and sciatic nerve blocks are especially effective for anesthesia and postoperative pain management in knee, ankle, and foot surgeries, providing stable hemodynamics and extended pain relief without the complications of neuraxial methods [13]. Ropivacaine, a long-acting amide local anesthetic, is widely used for its favorable pharmacological properties. As the pure S(-)-enantiomer, it selectively inhibits sodium ion influx in nerve fibers, primarily affecting nociceptive A-delta and C fibers over motor Aβ fibers, due to its lower lipophilicity compared with bupivacaine. This selective sensory blockade minimizes motor impairment and reduces the risk of central nervous system and cardiovascular toxicity [14]. The pharmacokinetics of ropivacaine are linear and proportional to the dose, with significant hepatic metabolism occurring primarily via CYP1A2-mediated aromatic hydroxylation and CYP3A4-mediated N-dealkylation [13]. Recent evidence shows that ropivacaine influences macrophage biology. In a rat sciatic nerve injury model, it enhanced axon regeneration by modulating Nav1.8-mediated macrophage signaling, directly aiding nerve repair [15]. This study offered the initial evidence suggesting that ropivacaine has the potential to actively shift macrophage phenotype towards a pro-reparative state. However, the precise polarization mechanisms and the signaling pathways involved remain to be fully elucidated [15]. Ropivacaine’s ability to influence macrophage polarization offers a promising therapeutic potential, suggesting it could be repurposed to aid inflammation resolution and tissue repair in macrophage-related conditions.

However, several critical knowledge gaps remain. Clinically, peripheral nerve block with ropivacaine is routinely applied for postoperative analgesia in extremity trauma, yet all prior studies regarding regional anesthesia in ACS are limited to pain control; whether PNB could exert direct disease-modifying, tissue-protective effects against ACS-related muscle injury independent of analgesia has never been experimentally investigated. Moreover, previous mechanistic work on ropivacaine immunomodulation was mainly established in peripheral nerve injury models, where ropivacaine promoted M2 macrophage transition via Nav1.8-related signaling [15]. Nevertheless, it remains unknown how ropivacaine reshapes macrophage polarization in the setting of ACS-triggered ischemia–reperfusion skeletal muscle injury, and whether distinct downstream signaling pathways are engaged in this pathological context.

Therefore, the present study addresses two interconnected scientific questions: (i) Does ropivacaine-based PNB (especially combined sciatic-femoral block) mitigate tissue damage, inflammation and macrophage dysregulation in experimental ACS, beyond analgesic action? (ii) What key signaling pathway mediates ropivacaine-induced M2 macrophage polarization under ACS-mimicking inflammatory conditions? To answer these questions, we combined an in vivo rat ACS model, in vitro RAW264.7 macrophage inflammatory model, RNA-seq transcriptome profiling, and pharmacological rescue experiments using the JAK2-specific inhibitor AG490.

## 2. Materials and Methods

### 2.1. Animals

Adult male Sprague-Dawley rats (6–8 weeks old, 180–220 g), free of specific pathogens, were obtained from Guizhou Medical University’s Experimental Animal Center. All animals were reared in a standardized laboratory environment with constant temperature (22 ± 2 °C), relative humidity of 50–60%, and a 12 h light-dark cycle. Standard rodent feed and sterile drinking water were provided ad libitum. All rats underwent a 7-day adaptive feeding period prior to formal experiments. All animal experimental procedures were approved by the Institutional Animal Care and Use Committee of Beijing Jishuitan Hospital Guizhou Hospital and complied with the Laboratory Animal Care Guidelines issued by the National Institutes of Health (NIH).

A closed soft tissue compression method was adopted to establish the ACS model. Briefly, rats were intraperitoneally injected with 50 mg/kg sodium pentobarbital for anesthesia, and successful anesthesia was confirmed by the disappearance of the toe-pinch reflex. A self-made pressure device was used to apply continuous 4.5 kg mechanical compression unilaterally to the anterolateral compartment of the right hindlimb for 2 h. After compression, the pressure clamp was removed to achieve tissue reperfusion. This modeling method can stably induce increased intracompartmental pressure, inflammatory cell infiltration and progressive skeletal muscle injury, which are consistent with the typical pathological characteristics of ACS.

Rats were randomly divided into two experimental cohorts via a computer-generated random sequence, with 6 rats in each group. Cohort 1 was designed to compare the therapeutic effects of different PNB modes, including five groups: sham operation group (Control), ACS model group, ACS + sciatic nerve block group, ACS + femoral nerve block group, and ACS + combined sciatic-femoral nerve block group. Cohort 2 was set for mechanistic exploration, containing four groups: Control group, ACS model group, ACS + ropivacaine combined nerve block group, and ACS + ropivacaine + AG490 intervention group. In the AG490 intervention group, the JAK2-specific inhibitor AG490 (Selleck Chemicals, Houston, TX, USA) was intraperitoneally administered at a daily dose of 10 mg/kg, with the first injection performed 30 min before ropivacaine treatment.

The sample size of each group was determined by G*Power 3.1.9.7 software based on preliminary experimental data, with an effect size of 0.8, an α value of 0.05, and a test power of 0.80, confirming n = 6 for each group. At the conclusion of the experiment, rats were euthanized by administering a high dose of sodium pentobarbital (150 mg/kg) intraperitoneally, followed by exsanguination.

### 2.2. PNB Operation

The rats were anesthetized with 50 mg/kg pentobarbital sodium and placed prone. The right thigh’s dorsal skin was shaved and disinfected with povidone-iodine. A 2 cm incision near the femur allowed blunt dissection of the biceps femoris to expose the sciatic nerve. Ropivacaine hydrochloride (Zhejiang Xianju Pharmaceutical Co., Ltd., Taizhou, China) was diluted with sterile saline to a 0.5% solution. Under a surgical microscope (Leica, Germany), 0.2 mL ropivacaine solution was evenly injected around the sciatic nerve using a 30-gauge Hamilton syringe, and the incision was sutured with 4-0 silk thread.

Subsequently, rats were fixed in a supine position, and the skin of the anterior thigh was disinfected routinely. A 1.5 cm incision was made at the femoral triangle to expose the femoral nerve, which was carefully isolated to avoid vascular damage. A total of 0.15 mL of 0.5% ropivacaine solution was injected circumferentially around the femoral nerve, followed by incision suture.

For the combined nerve block group, the above two operations were completed successively in one session, with a total ropivacaine dosage of 0.35 mL (1.75 mg per rat). All PNB treatments were performed immediately after the 2 h compression and clamp removal. The nerve block intervention was repeated daily for a consecutive 5-day observation period. Behavioral and functional indices were assessed on days 0, 1, 2, 3, 4 and 5 after modeling.

### 2.3. Functional Assessments

The body weight of rats was measured by a high-precision electronic balance (accuracy: 0.1 g, Mettler Toledo, Greifensee, Switzerland) before modeling (baseline) and on days 1–5 after modeling. The limb circumference of the right hindlimb was measured at the fixed midpoint between the ankle and knee joints with a flexible tape (accuracy: 0.5 mm) to evaluate limb swelling.

The thermal pain threshold of rats was tested by the Hargreaves thermal radiation method (Ugo Basile, Gemonio, Italy). After 15 min of adaptation on a glass test platform, thermal radiation with an intensity of 60 arbitrary units was applied to the plantar surface of the right hindlimb, and the paw withdrawal latency (PWL) was automatically recorded. A 45 s cut-off time was set to avoid tissue burn. Each rat was tested three times at 5 min intervals, and the average value was taken for statistical analysis.

Needle manometry was used to detect intracompartmental pressure. Briefly, rats were anesthetized with 2% isoflurane, and a 22-gauge needle connected to a pressure transducer (Abbott Laboratories, Abbott Park, IL, USA) was inserted 5 mm deep into the tibialis anterior muscle compartment. After 30 s of stable pressure balance, the compartment pressure data were collected via the PowerLab acquisition system (ADInstruments, Bella Vista, Australia).

### 2.4. Histological Analysis

Rat tibialis anterior muscle tissues were dissected and fixed in 4% paraformaldehyde solution for 48 h. After gradient ethanol dehydration, xylene clearing, and paraffin embedding, continuous 5 μm transverse tissue sections were prepared using a paraffin slicer Leica RM2235 (Leica, Nussloch, Germany) and mounted on glass slides for staining.

Hematoxylin-eosin (HE) staining was performed to observe the general morphological structure and inflammatory injury of muscle tissues. Masson’s trichrome staining (Sigma-Aldrich kit) and Sirius Red staining were used to label and observe collagen fiber deposition in muscle tissues. All staining operations were carried out in strict accordance with the kit instructions. After staining, the sections were sealed and photographed under an optical microscope (Olympus BX53, Olympus Corporation, Hachioji, Japan) at 200× magnification.

The pathological injury of HE-stained sections was evaluated by two independent observers blinded to group allocation using a 0–3 grading standard: 0 points for normal tissue structure, 1 point for mild inflammatory infiltration, 2 points for moderate inflammation with focal muscle necrosis, and 3 points for severe inflammatory response and extensive necrotic lesions. Formal inter-observer agreement statistics were not acquired; any scoring discrepancies between the two observers were resolved by consensus discussion. ImageJ software (v1.53) was used to quantify the percentage of collagen-positive area in five random visual fields of each section to evaluate the degree of tissue fibrosis. ImageJ software (v1.53) was used to quantify the percentage of collagen-positive area in five random visual fields of each section. Quantitative values obtained from five fields of the same section/animal were averaged to yield one value per biological replicate prior to statistical analysis.

### 2.5. Cell Culture and Treatment

Mouse RAW264.7 macrophage cell line (ATCC TIB-71, ATCC, Manassas, VI, USA) was cultured in high-glucose DMEM medium (Gibco, Grand Island, NY, USA) supplemented with 10% fetal bovine serum, 100 U/mL penicillin and 100 μg/mL streptomycin. The cells were incubated in a constant-temperature incubator at 37 °C and 5% CO_2_. When the cell confluence reached 80–90%, subculture was performed every 2–3 days. All cells were cultured overnight to adhere to the wall before subsequent experimental intervention.

RAW264.7 cells were induced by combined stimulation with 100 ng/mL LPS (Sigma-Aldrich, St. Louis, MO, USA) and 20 ng/mL IFN-γ (R&D Systems, Minneapolis, MN, USA) for 24 h to construct an inflammatory macrophage model. Of note, this is a simplified inflammatory model and does not fully recapitulate the complex ischemia–reperfusion-related microenvironment of ACS. To screen the optimal drug concentration, different concentrations of ropivacaine (1, 2.5, 5, 10 μg/mL) were added simultaneously with LPS/IFN-γ stimulation. The 5 μg/mL ropivacaine, with optimal intervention effect, was selected for follow-up experiments, and cells were pretreated with ropivacaine for 30 min before LPS/IFN-γ stimulation.

AG490 powder was dissolved in DMSO to prepare a 10 mM stock solution and stored at −20 °C. The working concentration of AG490 was set to 10 μM, and cells were pretreated with AG490 for 1 h before ropivacaine and inflammatory factor stimulation. The final DMSO concentration in all cell culture systems was controlled below 0.1% to avoid solvent toxicity. The cell experiment was divided into four groups: blank control group, LPS + IFN-γ model group, LPS + IFN-γ + ropivacaine intervention group, and LPS + IFN-γ + ropivacaine + AG490 co-intervention group.

### 2.6. Cell Functional Assays

CCK-8 assay: RAW264.7 cells were inoculated into 96-well plates at a density of 5 × 10^3^ cells per well. After adherence, cells were treated with different concentrations of ropivacaine combined with or without LPS/IFN-γ stimulation for 24 h. Subsequently, 10 μL CCK-8 reagent (Dojindo, Kumamoto, Japan) was added to each well, and the cells were incubated at 37 °C for 2 h. The absorbance value at 450 nm was detected by a microplate reader, and cell viability was calculated with the blank control group as the reference.

Wound-healing assay: Cells were seeded into 6-well plates at 2 × 10^5^ cells per well and cultured to full confluence. A uniform scratch was made on the cell monolayer with a sterile 200 μL pipette tip. Floating and detached cells were washed away with PBS, and serum-free DMEM medium containing the corresponding drugs was added for continuous culture. Cell scratch images were captured at 0 h and 24 h under an inverted microscope (100× magnification, Olympus IX73). The scratch closure rate was calculated via ImageJ software according to the formula: (0 h scratch area–24 h scratch area)/0 h scratch area × 100%.

Transwell migration assay: A total of 5 × 10^4^ RAW264.7 cells suspended in 200 μL serum-free DMEM were seeded into the upper chamber of 8 μm Transwell inserts (Corning, NY, USA). The lower chamber was filled with 600 μL DMEM medium containing 10% fetal bovine serum and corresponding intervention drugs. After 24 h of conventional culture, the non-migrated cells on the upper membrane were wiped off. The migrated cells on the lower membrane were fixed with 4% paraformaldehyde for 15 min and stained with 0.1% crystal violet for 20 min. After cleaning and drying, five random visual fields were photographed under a 200× microscope, and the number of migrated cells was counted statistically.

### 2.7. RNA Sequencing

Total RNA was extracted from LPS/IFN-γ-stimulated RAW264.7 macrophage cells using TRIzol reagent (Invitrogen, Carlsbad, CA, USA). RNA purity and integrity were detected by NanoDrop 2000 spectrophotometer (A260/A280 = 1.8–2.1) and an Agilent 2100 Bioanalyzer (RIN ≥ 7.0), Santa Clara, CA, USA. Qualified RNA samples were used to construct stranded mRNA libraries with the TruSeq Stranded mRNA Library Prep Kit (Illumina), and high-throughput sequencing was performed on the NovaSeq 6000 platform (sequencing mode: 2 × 150 bp, data volume ≥6 Gb per sample). The raw sequencing reads were subjected to quality control by FastQC v0.11.9, and adapter sequences and low-quality reads were trimmed by Trimmomatic v0.39. The clean reads were aligned to the mouse reference genome mm10 (GRCm38) using HISAT2 v2.2.1 software. FeatureCounts v2.0.1 was used to calculate gene expression counts. Differential expression analysis of genes was completed via the DESeq2 package (v1.38.0) in R 4.2.1 software. Genes with adjusted *p* < 0.05 and |log2FC| ≥ 1 were defined as significantly differentially expressed genes. The clusterProfiler v4.6.0 package was used for KEGG pathway enrichment analysis [16], and *p* < 0.05 after Benjamini–Hochberg correction was regarded as statistically significant. RNA-seq was performed with n = 3 independent biological replicates (independent cell culture batches) per experimental group.

### 2.8. Western Blot

Tissues and cells were lysed in RIPA buffer (Beyotime Biotechnology, Shanghai, China) with 1% PMSF and phosphatase inhibitors (Sigma-Aldrich). Lysates were centrifuged (12,000× *g*, 15 min, 4 °C) and protein concentrations were determined by BCA assay (Beyotime). Protein (30 μg/lane) was separated by 10% SDS-PAGE (120 V, 90 min) and transferred to PVDF membranes (0.45 μm; Millipore, 300 mA, 90 min, Burlington, MA, USA). Membranes were blocked with 5% non-fat milk in TBST (1 h), then incubated overnight at 4 °C with primary antibodies: p-JAK2 (Tyr1007/1008; 1:1000; CST #3771, Danvers, MA, USA), JAK2 (1:1000; CST #3230), p-STAT3 (Tyr705; 1:1000; CST #9145), STAT3 (1:1000; CST #4904), CD86 (1:500; Abcam ab112490, Abcam, Cambridge, UK), CD206 (1:500; Abcam ab64693), iNOS (1:500; Abcam ab178945), Arg-1 (1:500; Abcam ab124917), IL-6 (1:500; Abcam ab6672), CRP (1:500; Abcam ab31156), and β-actin (1:5000; Sigma A5316). HRP-conjugated secondary antibodies (1:5000; Jackson ImmunoResearch, West Grove, PA, USA) were applied for 1 h at room temperature. Bands were detected by ECL (Millipore) and imaged on a ChemiDoc XRS+ (Bio-Rad, Hercules, CA, USA). Densitometry was performed using ImageJ, normalized to β-actin. Phosphorylated proteins were additionally normalized to their total protein counterparts.

### 2.9. Immunohistochemistry (IHC)

Paraffin tissue sections (5 μm) were deparaffinized and rehydrated, followed by antigen retrieval with sodium citrate buffer (10 mM, pH 6.0) at 95 °C for 20 min. Endogenous peroxidase activity was blocked with 3% hydrogen peroxide-methanol solution for 15 min, and non-specific binding sites were sealed with 5% normal goat serum for 30 min. The sections were incubated with primary antibodies (IL-6, CRP, p-JAK2, JAK2, p-STAT3, STAT3) overnight at 4 °C.

After washing, biotin-labeled goat anti-rabbit secondary antibody and ABC HRP kit were used for incubation in sequence. DAB chromogenic solution was applied for color development, and hematoxylin was used for nuclear counterstaining. After dehydration and clearing, the sections were sealed and photographed. Five random visual fields of each section (200×) were analyzed by the ImageJ-IHC Profiler plugin to quantify staining intensity and positive area. Measurements from multiple fields derived from the same biological sample were averaged to produce a single data point per animal before statistical comparison. The negative control group was set without primary antibody incubation.

### 2.10. Immunofluorescence (IF) Staining

For tissue immunofluorescence detection, paraffin sections were processed with antigen retrieval referring to the immunohistochemical procedure, then blocked with 5% goat serum containing 0.3% Triton X-100 for 1 h. For cell immunofluorescence, RAW264.7 cells on coverslips were fixed with 4% paraformaldehyde for 15 min, permeabilized with 0.3% Triton X-100 for 10 min, and blocked with 5% goat serum for 1 h. All samples were incubated with primary antibodies (CD86, CD206, iNOS, Arg-1, p-JAK2, JAK2, p-STAT3, STAT3; all 1:200) overnight at 4 °C. After washing, Alexa Fluor 488 fluorescent secondary antibody was added for 1 h of dark incubation. Nuclei were stained with DAPI working solution for 10 min. Finally, the samples were sealed with an anti-fluorescence-quenching mounting medium. A confocal laser scanning microscope (Zeiss LSM 880, Carl Zeiss Microscopy, Jena, Germany) was used for imaging, with 200× magnification for tissue samples and 400× magnification for cell samples. ImageJ software was used to detect the mean fluorescence intensity of five random visual fields per sample. Fluorescence intensity values across five fields from one biological sample were averaged, generating one independent value per animal/sample for subsequent statistical analysis.

### 2.11. ELISA

Blood from rats was obtained via cardiac puncture, and serum was isolated by centrifuging at 3000× *g* for 15 min at 4 °C. Fresh muscle tissues were homogenized in ice-cold PBS containing protease inhibitors, and the supernatant was collected after centrifugation at 12,000× *g* for 15 min. The expression levels of IL-6 and CRP in serum and tissue homogenate were detected using commercial rat-specific ELISA kits in strict accordance with the manufacturer’s instructions. The absorbance at 450 nm was measured by a Tecan Infinite M200 microplate reader. The standard curve was fitted to calculate the sample protein concentration, and the unit was expressed as pg/mL (IL-6) and ng/mL (CRP).

### 2.12. Quantitative Real-Time PCR (qRT-PCR)

Total tissue RNA was extracted using TRIzol reagent and quantified by NanoDrop 2000 spectrophotometer (Thermo Fisher Scientific, Wilmington, DE, USA). A total of 1 μg of qualified RNA was reverse-transcribed into cDNA using the PrimeScript RT reagent kit with gDNA eraser (Takara Bio, Kusatsu, Japan). The qRT-PCR reaction was performed with TB Green Premix Ex Taq II kit on a QuantStudio 3 PCR system. The 20 μL reaction system included 10 μL TB Green Premix, 0.8 μL forward and reverse primers (10 μM), respectively, 2 μL cDNA template and 6.4 μL nuclease-free water. The PCR amplification program was set as follows: pre-denaturation at 95 °C for 30 s, followed by 40 cycles of 95 °C for 5 s and 60 °C for 30 s. Melting curve analysis was performed to verify the specificity of amplification products. β-actin was used as the internal reference gene. The relative mRNA expression levels of TNF-α, IL-6, IL-1β and CRP were calculated by the 2^−ΔΔCt^ method, and each sample was tested in triplicate.

Primer sequences are shown as follow: TNF-α (Forward: GACGTGGAACTGGCAGAAGAG; Reverse: TTGGTGGTTTGTGAGTGTGAG), IL-6 (Forward: GCTACCAAACTGGATATAATCAGGA; Reverse: CCAGGTAGCTATGGTACTCCAGAA), IL-1β (Forward: CACAGCAGCATCTCGACAAG; Reverse: GTGCTGCCTAATGTCCCCTT), CRP (Forward: TGACGTCTCTGGGCAAGGATTG; Reverse: CCGAAGAGGATTCCATAGCAGG), β-actin (Forward: CATGTACGTTGCTATCCAGGC; Reverse: CTCCTTAATGTCACGCACGAT).

### 2.13. Statistical Analysis

Definition of replicates: All n values shown in figure legends correspond to independent biological replicates. For in vivo experiments, n = 6 means six individual rats for functional assays, histological staining, ELISA and immunofluorescence; n = 3 for Western blot indicates three independent individual rats, reduced due to limited protein amount isolated from each muscle specimen. Technical replicates (e.g., qPCR technical triplicates) were averaged within each biological sample and were not counted as independent n in statistical tests. For all microscopy-related quantifications, multiple fields from the same biological sample were averaged to obtain one data point per biological replicate before group comparison; individual fields were not treated as independent replicates.

All experimental data were analyzed by GraphPad Prism 9.0 and SPSS 26.0, and the results were presented as mean ± standard deviation (mean ± SD). The Shapiro–Wilk test and Levene’s test were used to verify the normality of data distribution and homogeneity of variance, respectively. An independent-samples t-test was used for two-group comparisons. For multi-group comparisons, one-way ANOVA followed by Tukey’s HSD post hoc test for pairwise multiple-comparison correction was performed to control type I error. Longitudinal repeated-measures data (body weight, thermal withdrawal latency, limb circumference, intracompartmental pressure) were analyzed using repeated-measures two-way ANOVA with Bonferroni post hoc correction for multiple comparisons across time points and groups. Significance asterisks in figures represent corrected post hoc pairwise comparisons (* *p* < 0.05, ** *p* < 0.01, *** *p* < 0.001). Raw adjusted pairwise *p*-value outputs are archived and available from the corresponding author upon reasonable request. *p* < 0.05 was defined as statistically significant. All in vitro and in vivo experiments were repeated for no less than 3 independent biological replicates. Animals were randomly allocated into experimental groups shortly before ACS induction. The researcher who performed randomization and prepared the treatment solutions was not involved in subsequent data collection or outcome evaluation. All investigators performing functional tests, histological scoring and laboratory measurements remained blinded to group-allocation information throughout data acquisition to avoid subjective bias. For histological injury scoring, two independent blinded observers performed scoring, with discrepancies resolved by consensus.

## 3. Results

### 3.1. Peripheral Nerve Block Ameliorates ACS-Induced Injury in Rats

**Figure 1:**
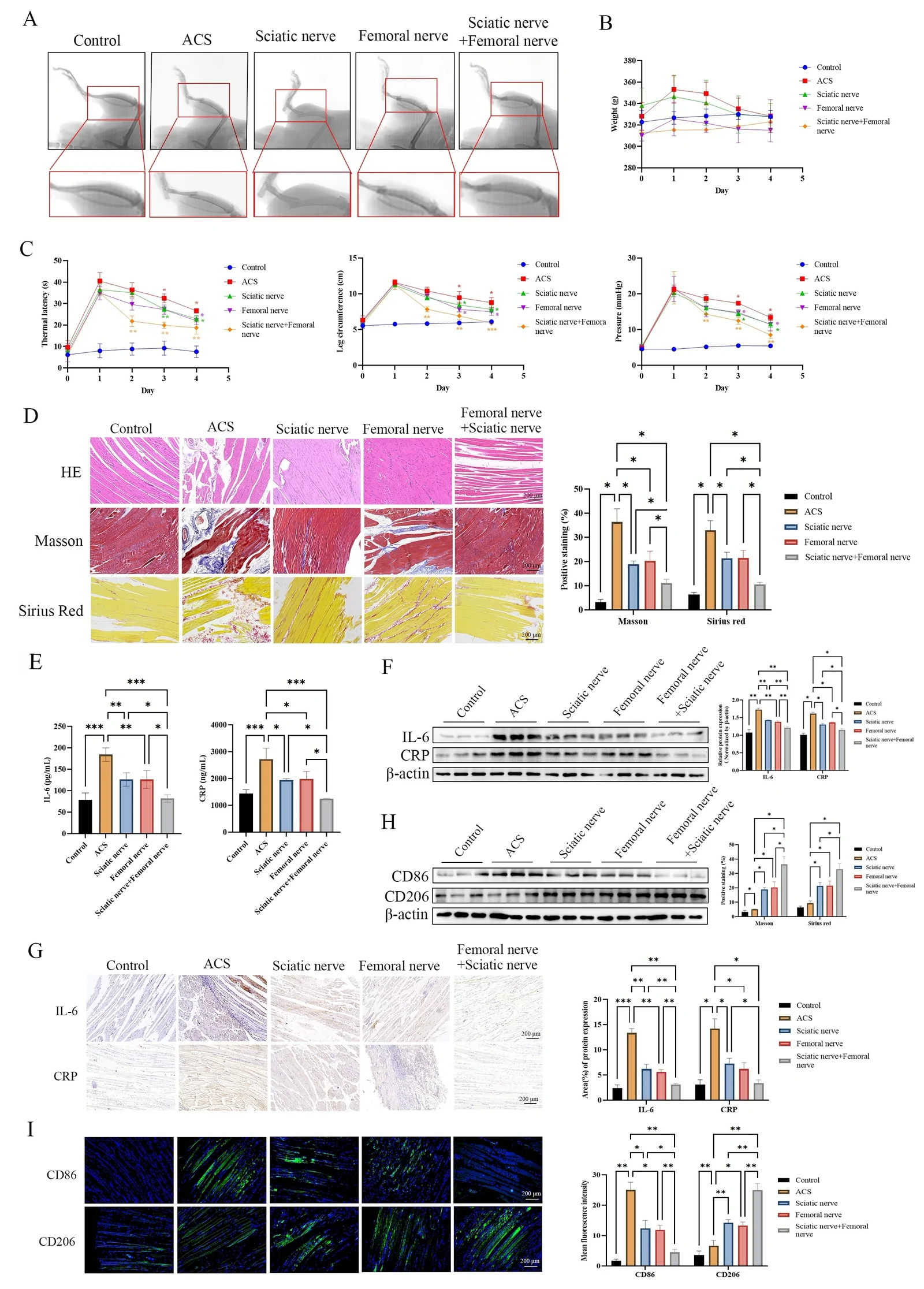
Peripheral nerve block (PNB) with ropivacaine ameliorates acute compartment syndrome (ACS)-induced injury in rats and promotes macrophage M2 polarization. (**A**) Representative photographs of unilateral injured right hindlimb from the Control, ACS, sciatic nerve block, femoral nerve block, and combined (sciatic + femoral) nerve block groups, showing gross morphological changes. Enlarged views highlight differences in tissue swelling and discoloration among groups. (**B**) Body weight changes monitored over 5 days post-ACS induction (n = 6 per group, biological replicates). (**C**) Functional assessments over the 5-day observation period, including thermal withdrawal latency (**left**), leg circumference (**middle**), and intracompartmental pressure (**right**) (n = 6 per group, biological replicates). Error bars represent standard deviation (SD). * *p* < 0.05, ** *p* < 0.01, *** *p* < 0.001 versus the ACS group. (**D**) Representative histological staining of tibialis anterior muscle sections. Hematoxylin-eosin (HE) staining shows general tissue architecture; Masson’s trichrome staining reveals collagen deposition (blue); Sirius Red staining demonstrates collagen fibers under polarized light (red). Scale bar = 200 μm. Right panel: semiquantitative analysis of Masson- and Sirius Red-positive staining areas (n = 6 per group, biological replicates). (**E**) Serum levels of interleukin-6 (IL-6) and C-reactive protein (CRP) measured by ELISA on day 5 (n = 6 per group, biological replicates). (**F**) Western blot analysis of IL-6 and CRP expression in muscle tissue, with β-actin as a loading control. Right panel: densitometric quantification (n = 3 per group, biological replicates). (**G**) Representative immunohistochemistry (IHC) images of IL-6 and CRP in muscle tissue sections. Scale bar = 200 μm. Right panel: semiquantitative analysis of IHC staining intensity (n = 6 per group, biological replicates). (**H**) Western blot analysis of macrophage polarization markers CD86 (M1) and CD206 (M2) in muscle tissue, with β-actin as loading control. Right panel: densitometric quantification (n = 3 per group, biological replicates). (**I**) Representative immunofluorescence (IF) staining of CD86 and CD206 in muscle tissue cryosections. Scale bar = 200 μm. Right panel: quantification of mean fluorescence intensity (n = 6 per group, biological replicates). Error bars represent standard deviation (SD). * *p* < 0.05, ** *p* < 0.01, *** *p* < 0.001.

To determine the therapeutic benefits of PNB in ACS, we used a rat model with ACS induced unilaterally in the right hindlimb. Animals received sciatic nerve block, femoral nerve block, or combined sciatic-femoral nerve block using ropivacaine. Throughout the five-day observation, macroscopic analysis of the lower limbs revealed marked differences among the experimental groups (Figure 1A). Leg swelling and evident discoloration were observed in rats from the ACS group, highlighting severe tissue damage and inflammation-related edema. In contrast, animals treated with PNB showed milder pathological changes over time, with the combined nerve block group closely resembling the control group in appearance.

The measurement of body weight changes quantitatively underscored the protective benefits of PNB intervention. Control animals maintained stable weights around 330 g. The ACS group initially lost weight but partially recovered. Rats with sciatic or femoral nerve blocks showed moderate weight maintenance, while those with combined nerve blocks had the best recovery, nearly reaching control weights by the study’s end (Figure 1B).

The thermal latency test revealed notable differences in pain responses among groups. Control rats withdrew quickly (~10 s), whereas ACS-group rats took much longer (~45 s), indicating sensory dysfunction. PNB-treated groups had shorter latency times than the ACS group, with the combined nerve block showing the most normal results, suggesting that PNB treatment effectively reduces ACS-induced sensory impairment. (Figure 1C, left panel).

Additional evidence for the protective effects of PNB was found through physical measurements of leg circumference and compartment pressure (Figure 1C, middle and right panels). The ACS group showed a notable increase in leg circumference (about 12 mm vs. 5 mm in the control group) and compartment pressure (about 25 mmHg vs. 5 mmHg in the control group), confirming successful induction of compartment syndrome. PNB treatment significantly reduced both measures, with the combined block providing the greatest reduction. This suggests that PNB effectively alleviates the edema and pressure increase linked to ACS.

The tibialis anterior muscle sections were histologically examined using Hematoxylin and Eosin (HE) staining, Masson’s trichrome staining, and Sirius Red staining to assess tissue structure, collagen buildup, and fibrotic changes, respectively (Figure 1D). HE staining showed severe muscle fiber damage in the Acute Compartment Syndrome (ACS) group, with inflammatory cell infiltration, necrotic fibers, and disrupted striation patterns. Groups treated with Peripheral Nerve Block (PNB) showed progressive improvement in histopathological damage, with the combined block group having muscle fiber architecture similar to control tissue. Masson’s trichrome staining revealed significant collagen deposition, indicating severe fibrosis, which was supported by Sirius Red staining showing abundant collagen fibers. Quantitative analysis showed that PNB treatment significantly reduced both Masson-positive and Sirius Red-positive staining, with the combined nerve block achieving the lowest fibrosis levels among all groups.

The assessment of systemic and local inflammatory responses was conducted by measuring IL-6 and CRP levels using ELISA (Figure 1E). Acute compartment syndrome (ACS) induction resulted in a notable rise in IL-6 and CRP levels compared with control values, suggesting a robust inflammatory reaction to compartment injury. There was a notable decline in circulating inflammatory marker levels across all peripheral nerve block (PNB) treatment groups. Among the three PNB regimens, combined sciatic-femoral blockade produced the largest decrease in IL-6 and CRP levels. These findings were corroborated by analyzing muscle tissue lysates using Western blot (Figure 1F), The ACS group exhibited strong IL-6 and CRP immunoreactive bands, which were significantly reduced in all PNB treatment groups. Immunohistochemical staining showed intense IL-6 and CRP immunoreactivity in the ACS group, with a gradual decrease in the sciatic, femoral, and combined block groups. Statistical analysis confirmed significant reductions in IL-6 and CRP immunoreactivity following PNB intervention.

Given the critical role of macrophage polarization in inflammation resolution and tissue repair, we detected well-established macrophage phenotype markers (Figure 1H,I). Western blot results showed that the ACS group exhibited higher expression of the M1 marker CD86 and lower expression of the M2 marker CD206. The combined nerve-block group displayed the strongest shift toward an M2-like phenotype, with CD206 restored nearly to control levels and CD86 markedly suppressed. Immunofluorescence staining of muscle cryosections further confirmed these changes: ACS lesions showed intense CD86 but weak CD206 signals, while PNB treatment down-regulated CD86 and up-regulated CD206. We next established an in vitro M1-polarization model by stimulating RAW264.7 macrophages with LPS and IFN-γ to explore the direct effects of ropivacaine on macrophage phenotypes and the underlying molecular mechanisms.

### 3.2. Ropivacaine Promotes M2 Polarization and Inhibits Macrophage Migration

**Figure 2:**
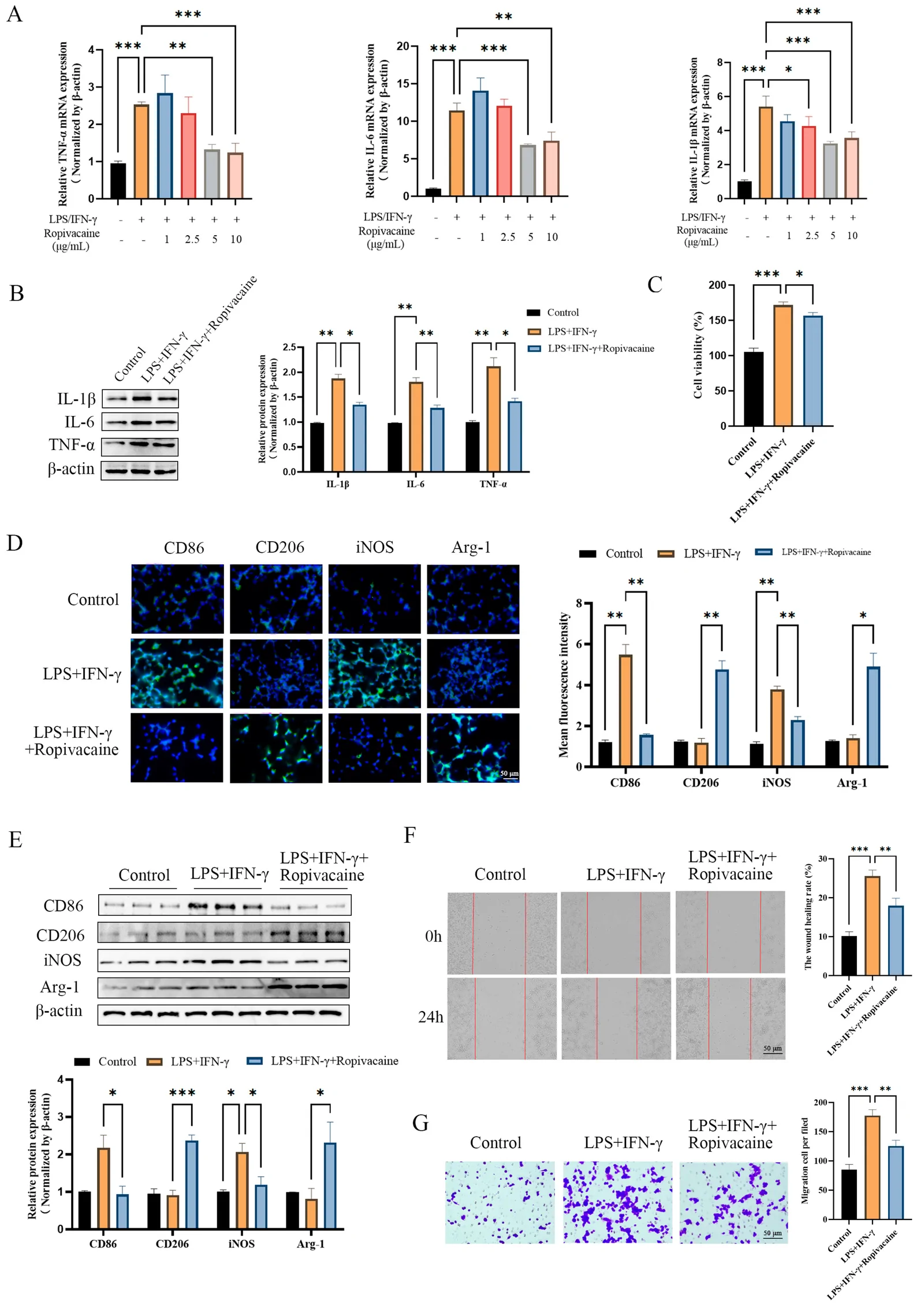
Ropivacaine promotes M2 macrophage polarization and inhibits macrophage migration in vitro. RAW264.7 macrophages were stimulated with lipopolysaccharide (LPS, 100 ng/mL) plus interferon-gamma (IFN-γ, 20 ng/mL) to induce M1 polarization, with or without ropivacaine treatment. (**A**) Dose–response analysis of ropivacaine (1, 2.5, 5, and 10 μg/mL) on the mRNA expression of pro-inflammatory cytokines TNF-α, IL-6, and IL-1β, as determined by quantitative real-time PCR (qRT-PCR) (n = 3 independent experiments). (**B**) Western blot analysis of IL-1β, IL-6, and TNF-α protein expression, with corresponding densitometric quantification (n = 3, biological replicates). (**C**) Cell viability assessed by Cell Counting Kit-8 (CCK-8) assay following ropivacaine (5 μg/mL) treatment for 24 h (n = 6, biological replicates). (**D**) Representative immunofluorescence images and quantification of M1 markers (CD86, iNOS) and M2 markers (CD206, Arg-1). Scale bar = 50 μm (n = 6, biological replicates). (**E**) Western blot analysis of CD86, CD206, iNOS, and Arg-1 protein expression, with corresponding densitometric quantification (n = 3, biological replicates). (**F**) Wound-healing (scratch) assay images captured at 0 and 24 h, with quantification of wound closure rates. Scale bar = 50 μm (n = 6, biological replicates). (**G**) Transwell migration assay images and quantification of migrated cells. Scale bar = 50 μm (n = 6, biological replicates). Error bars represent standard deviation (SD). * *p* < 0.05, ** *p* < 0.01, *** *p* < 0.001. Significance markers connecting the Control and LPS + IFN-γ groups indicate comparisons versus Control; markers connecting the LPS + IFN-γ and LPS + IFN-γ + ropivacaine groups indicate comparisons versus the LPS + IFN-γ group.

An in vitro model of M1 macrophage polarization was established by stimulating RAW264.7 cells with LPS and IFN-γ to investigate the direct cell-autonomous effects of ropivacaine on inflammatory macrophage polarization using a simplified LPS/IFN-γ-stimulated RAW264.7 cell model. Initially, a dose–response study was performed to find the best concentration of ropivacaine for influencing macrophage inflammatory reactions (Figure 2A). Macrophages were exposed to increasing doses of ropivacaine (1, 2.5, 5, and 10 μg/mL) alongside LPS and IFN-γ, and pro-inflammatory cytokine mRNA levels were measured via qRT-PCR. Ropivacaine at doses ≥ 2.5 μg/mL significantly lowered TNF-α, IL-6, and IL-1β expression compared with the LPS + IFN-γ group. The 5 μg/mL dose showed the most effective and consistent reduction in cytokine transcription, with significant decreases in TNF-α, IL-6, and IL-1β mRNA (*p* < 0.01, *p* < 0.001). Consequently, 5 μg/mL was chosen for future in vitro studies.

At the chosen optimal concentration, ropivacaine (5 μg/mL) was found to significantly reduce the transcription of all three pro-inflammatory cytokines in macrophages stimulated with LPS/IFN-γ (Figure 2A). TNF-α mRNA expression was markedly reduced compared with the stimulated control (*p* < 0.001), as were IL-6 (*p* < 0.01) and IL-1β (*p* < 0.05) transcript levels. Western blot analysis of whole-cell lysates (Figure 2B) showed that transcriptional changes were mirrored in protein levels. Densitometric analysis indicated that ropivacaine significantly reduced IL-1β, IL-6, and TNF-α protein expression compared with the LPS/IFN-γ-stimulated group, confirming ropivacaine’s suppression of pro-inflammatory cytokines at both transcriptional and translational stages.

To eliminate the potential confounding factor of ropivacaine-induced cytotoxicity in the observed anti-inflammatory effects, a Cell Counting Kit-8 (CCK-8) viability assay was conducted (Figure 2C). Macrophages were exposed to ropivacaine at a concentration of 5 μg/mL for the same duration as the functional experiments. According to the assay results, there was no notable reduction in cell viability when compared with untreated controls, indicating that the immunomodulatory effects of ropivacaine at this concentration are independent of nonspecific cytotoxicity.

The impact of ropivacaine on macrophage polarization was assessed using immunofluorescence for M1 and M2 markers. LPS/IFN-γ-stimulated macrophages showed strong CD86 and iNOS fluorescence, indicating an M1 pro-inflammatory phenotype, with low CD206 and Arg-1 staining, markers of the M2 anti-inflammatory phenotype. Ropivacaine treatment (5 μg/mL) significantly reduced CD86 and iNOS fluorescence while increasing CD206 and Arg-1 levels. Fluorescence intensity ratios confirmed significant changes in all four markers after ropivacaine exposure (Figure 2D).

These immunofluorescence observations were independently validated by Western blot analysis of M1 and M2 marker proteins (Figure 2E). Ropivacaine treatment notably reduced CD86 and iNOS protein levels while increasing CD206 and Arg-1 in LPS/IFN-γ-stimulated macrophages, aligning with fluorescence data. These changes were significant, with M2 markers nearing levels seen in unstimulated controls. Overall, ropivacaine facilitates a shift from an M1 pro-inflammatory to an M2 anti-inflammatory phenotype in these macrophages.

Besides affecting cytokine production and polarization marker expression, ropivacaine notably changed the movement patterns of activated macrophages in the wound-healing scratch test (Figure 2F); LPS/IFN-γ-stimulated macrophages quickly closed wounds within 24 h, showcasing their enhanced migratory ability typical of activated M1 macrophages. Conversely, ropivacaine-treated macrophages showed significantly reduced wound closure. Quantitative analysis confirmed that ropivacaine significantly hindered macrophage migration compared with stimulated controls. Ropivacaine’s ability to suppress macrophage migration was additionally supported by results from a Transwell migration assay (Figure 2G). Macrophages stimulated with LPS/IFN-γ exhibited significant movement through a porous membrane in response to a chemotactic gradient. Quantitative analysis confirmed that ropivacaine treatment greatly decreased cell migration. The findings reveal that ropivacaine supports M2 polarization, lowers pro-inflammatory cytokines, and restricts macrophage migration, suggesting a diverse immunomodulatory mechanism.

### 3.3. RNA-Seq Identifies JAK-STAT Signaling Pathway as a Key Mechanism Underlying Ropivacaine-Mediated Macrophage Polarization

**Figure 3:**
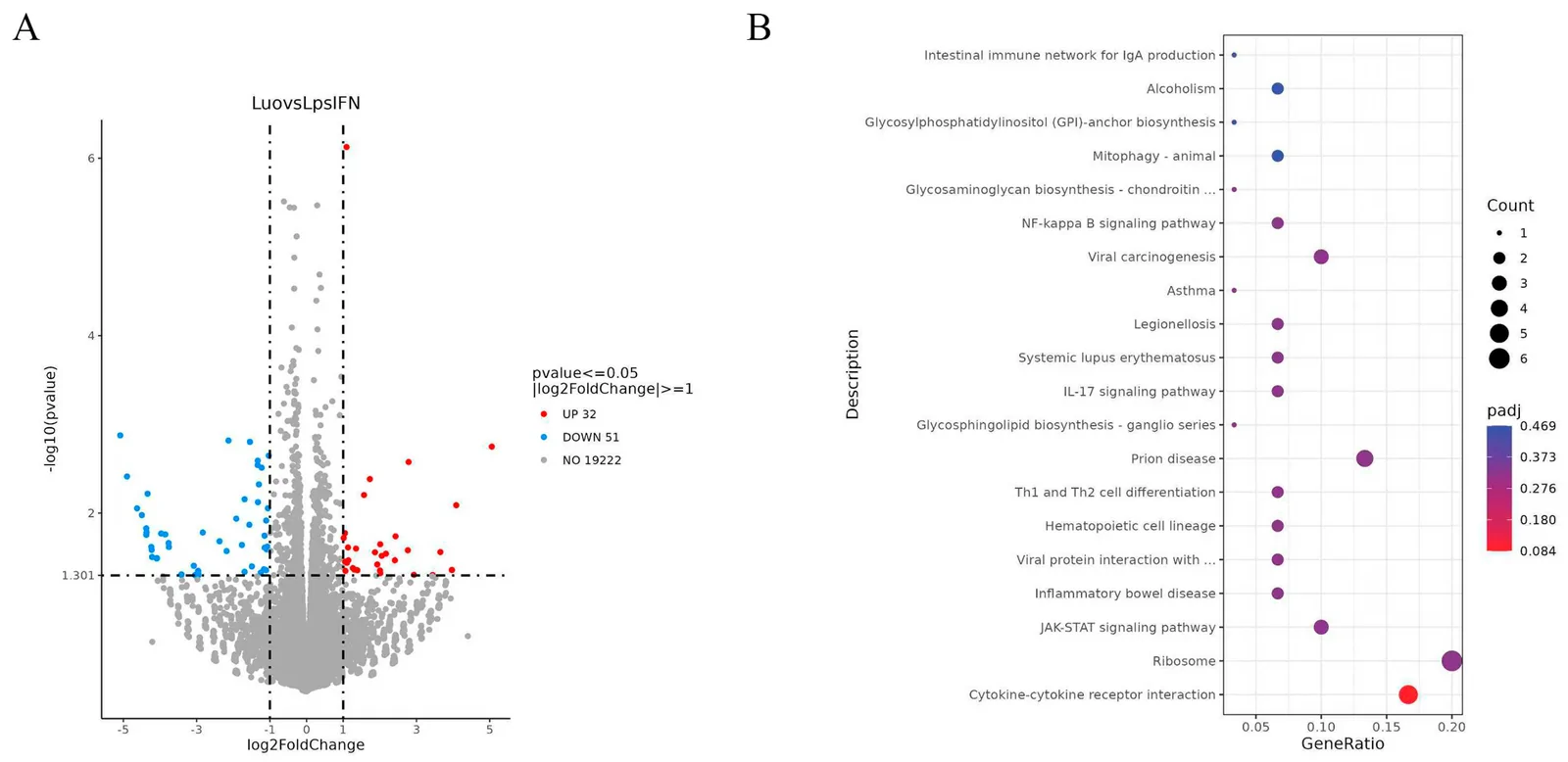
RNA sequencing identifies the JAK-STAT signaling pathway as a key mechanism underlying ropivacaine-mediated macrophage polarization. RNA-seq was performed on LPS + IFN-γ-stimulated RAW264.7 macrophages treated with or without ropivacaine (5 μg/mL) for 24 h (n = 3 per group, biological replicates). (**A**) Volcano plot showing differentially expressed genes (DEGs) between ropivacaine-treated and control-stimulated macrophages. Red dots indicate 32 upregulated genes (log_2_ fold change ≥ 1, adjusted *p* < 0.05); blue dots indicate 51 downregulated genes (log_2_ fold change ≤ −1, adjusted *p* < 0.05); gray dots represent 19,222 genes with no significant differential expression. Dashed lines indicate the thresholds for statistical significance (*p* = 0.05, horizontal) and fold change (|log_2_FC| = 1, vertical). (**B**) KEGG pathway enrichment analysis of the 83 DEGs. The JAK-STAT signaling pathway was identified as one of the most significantly enriched pathways. Bubble size represents gene count; color scale indicates adjusted *p*-value (padj), Pathway map was obtained from the KEGG database [16].

To explore how ropivacaine affects macrophage polarization at the transcriptome level, RNA-Seq was conducted on LPS/IFN-γ-stimulated RAW264.7 macrophages treated with or without 5 μg/mL ropivacaine for 24 h. Using strict criteria (absolute log2 fold change ≥ 1 and adjusted *p* < 0.05), 83 genes showed significant differential expression, with 32 upregulated (red) and 51 downregulated (blue) in the volcano plot (Figure 3A).

The clusterProfiler algorithm was used to perform KEGG pathway enrichment analysis on differentially expressed genes, with an adjusted *p*-value cutoff of 0.05, to pinpoint enriched biological pathways and functions (Figure 3B). The JAK-STAT signaling pathway was notably enriched among the differentially expressed genes, with many key regulatory components of this cascade present in the regulated transcripts. The JAK-STAT pathway showed strong enrichment, indicated by a high GeneRatio and low adjusted *p*-value, highlighting its role in macrophage polarization and cytokine signaling. This led us to focus on it as the main mechanism for ropivacaine-induced M2 polarization.

### 3.4. Ropivacaine Activates JAK-STAT Pathway to Promote M2 Polarization

**Figure 4:**
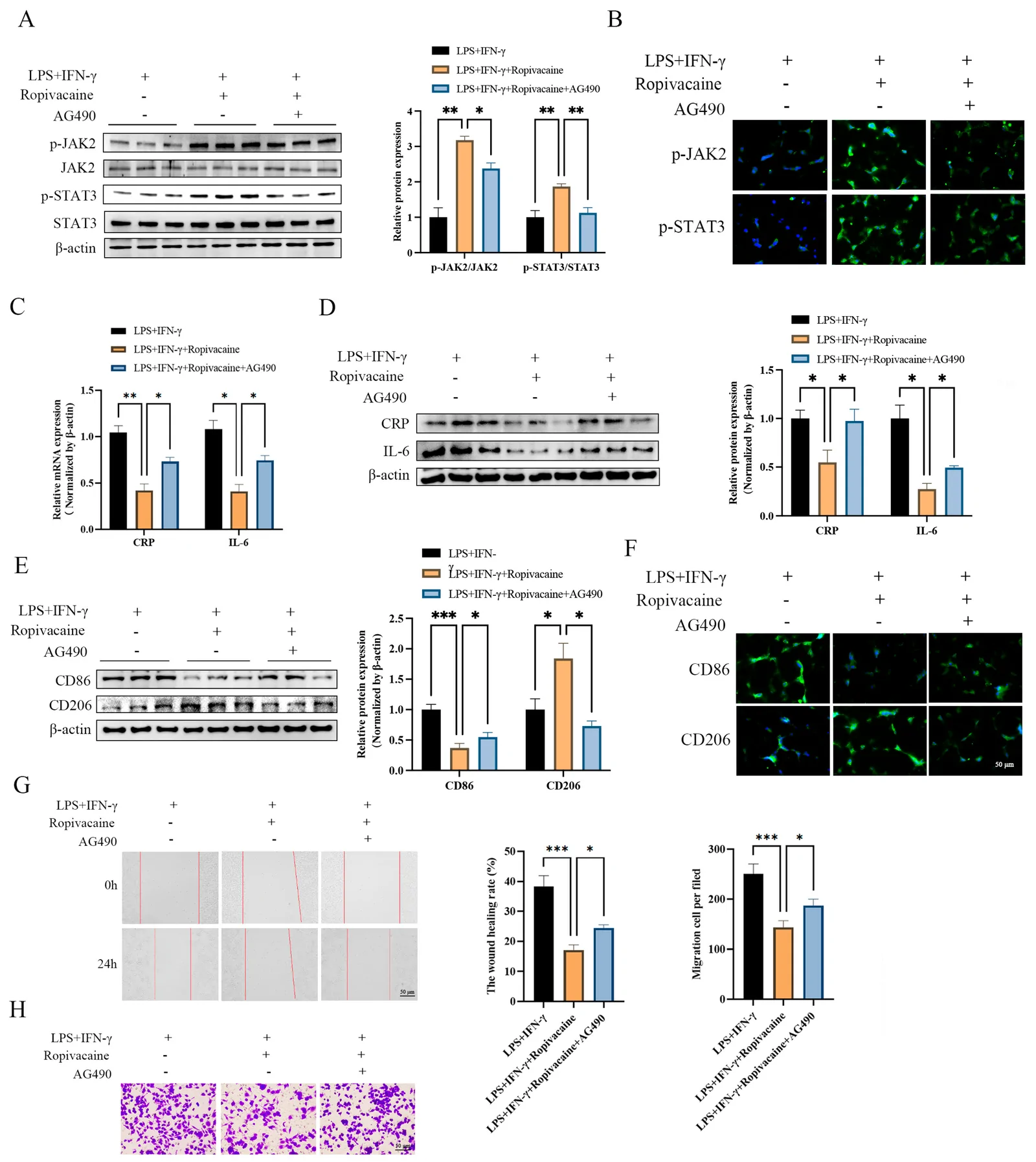
Ropivacaine promotes M2 macrophage polarization via activation of the JAK-STAT signaling pathway in vitro. LPS + IFN-γ-stimulated RAW264.7 macrophages were treated with ropivacaine (5 μg/mL) in the absence or presence of the selective JAK2 inhibitor AG490 (10 μM). (**A**) Western blot analysis of phosphorylated JAK2 (p-JAK2), total JAK2, phosphorylated STAT3 (p-STAT3), and total STAT3, with β-actin as a loading control. Right panel: densitometric quantification of p-JAK2/JAK2 and p-STAT3/STAT3 phosphorylation ratios (n = 3, biological replicates). (**B**) Representative immunofluorescence images and quantification of p-JAK2 and p-STAT3. Scale bar = 50 μm (n = 6, biological replicates). (**C**) Western blot analysis of CRP and IL-6 protein expression with corresponding densitometric quantification (n = 3, biological replicates). (**D**) qRT-PCR analysis of CRP and IL-6 mRNA expression (left panel), and immunofluorescence images and quantification of CRP and IL-6 protein expression (right panel). Scale bar = 50 μm (n = 6, biological replicates). (**E**) Western blot analysis of macrophage polarization markers CD86 and CD206 with corresponding densitometric quantification (n = 3, biological replicates). (**F**) Representative immunofluorescence images and quantification of CD86 and CD206. Scale bar = 50 μm (n = 6, biological replicates). (**G**) Wound-healing assay images at 0 and 24 h with quantification of wound closure rates (n = 6, biological replicates). (**H**) Transwell migration assay images and quantification of migrated cells. Scale bar = 50 μm (n = 6, biological replicates). Error bars represent standard deviation (SD). * *p* < 0.05, ** *p* < 0.01, *** *p* < 0.001.

To confirm the involvement of the JAK-STAT pathway in macrophage polarization caused by ropivacaine, Western blot analysis was performed to assess the phosphorylation levels of key elements in this pathway (Figure 4A). When stimulated with LPS/IFN-γ, macrophages demonstrated low phosphorylation levels of JAK2 and STAT3 under basic inflammatory conditions. However, treatment with ropivacaine (5 μg/mL) markedly elevated the phosphorylation of both JAK2 and STAT3. Densitometric analysis, normalized to total JAK2 and STAT3 protein levels, confirmed significant enhancements in the p-JAK2/JAK2 and p-STAT3/STAT3 ratios following ropivacaine treatment. To confirm JAK2’s role in the response, macrophages were treated with ropivacaine and AG490, a JAK2 inhibitor. AG490 fully blocked ropivacaine-induced JAK2 and STAT3 phosphorylation, returning their ratios to baseline levels. This shows that ropivacaine activates the JAK-STAT pathway through JAK2.

Next, IF staining was used to examine where activated JAK-STAT components are located within cells (Figure 4B). Consistent with the Western blot findings, ropivacaine treatment resulted in markedly enhanced nuclear immunofluorescence for p-JAK2 and p-STAT3, indicating ligand-independent activation and subsequent nuclear translocation of these transcriptional regulators. Total JAK2 and STAT3 proteins showed predominantly cytoplasmic distribution under all conditions, as expected. AG490 co-treatment effectively blocked the ropivacaine-induced nuclear accumulation of both p-JAK2 and p-STAT3, with fluorescent signals remaining predominantly cytoplasmic. Quantification of nuclear-to-cytoplasmic fluorescence intensity ratios confirmed statistically significant increases in nuclear p-JAK2 and p-STAT3 following ropivacaine treatment, which were completely prevented by JAK2 inhibition with AG490.

To determine whether JAK-STAT pathway activation was functionally required for the anti-inflammatory effects of ropivacaine, the expression of pro-inflammatory markers was assessed in the presence and absence of AG490 (Figure 4C,D). As observed previously, ropivacaine significantly reduced the protein and mRNA expression of CRP and IL-6 in LPS/IFN-γ-stimulated macrophages. However, co-administration of AG490 completely reversed these suppressive effects, restoring both CRP and IL-6 to levels statistically indistinguishable from those in ropivacaine-untreated stimulated controls. Western blot analysis of whole-cell lysates demonstrated restored CRP and IL-6 immunoreactive band intensities in the AG490 plus ropivacaine group compared with ropivacaine alone (Figure 4C), and qRT-PCR together with immunofluorescence staining independently confirmed the restoration of CRP and IL-6 mRNA and protein expression following JAK2 inhibition (Figure 4D). These data establish that JAK-STAT pathway activation is necessary for ropivacaine-mediated suppression of macrophage inflammatory responses.

The requirement for JAK-STAT signaling in ropivacaine-induced M2 polarization was further evaluated by examining macrophage phenotype markers following pathway inhibition (Figure 4E,F). Western blot analysis revealed that AG490 completely reversed the ropivacaine-induced shift in macrophage polarization marker expression (Figure 4E). In the presence of AG490, ropivacaine no longer suppressed CD86 expression or enhanced CD206 expression; instead, CD86 levels remained elevated and CD206 levels remained suppressed at magnitudes comparable to those observed in LPS/IFN-γ-stimulated macrophages without ropivacaine treatment. Immunofluorescence staining provided independent confirmation of these findings (Figure 4F), which demonstrated that AG490 inhibited the decrease in CD86 fluorescence and the increase in CD206 immunoreactivity caused by ropivacaine. Quantitative fluorescence analysis confirmed that JAK2 inhibition negated ropivacaine’s M2-promoting effects.

Ultimately, the impact of inhibiting the JAK-STAT pathway on macrophage movement was evaluated through wound-healing and Transwell assays (Figure 4G,H). Ropivacaine treatment significantly inhibited macrophage wound closure capacity and Transwell migration, as observed previously. AG490 co-treatment completely restored macrophage migratory behavior in both assays, with wound-healing rates and numbers of migrated cells returning to levels statistically comparable to those of LPS/IFN-γ-stimulated controls without ropivacaine. Collectively, these results demonstrate that ropivacaine promotes M2 macrophage polarization, suppresses pro-inflammatory responses, and inhibits macrophage migration through activation of the JAK-STAT signaling pathway, and that pharmacological blockade of JAK2 with AG490 is sufficient to reverse all of these functional effects.

### 3.5. JAK-STAT Signaling Mediates PNB-Induced Protective Effects in ACS

**Figure 5:**
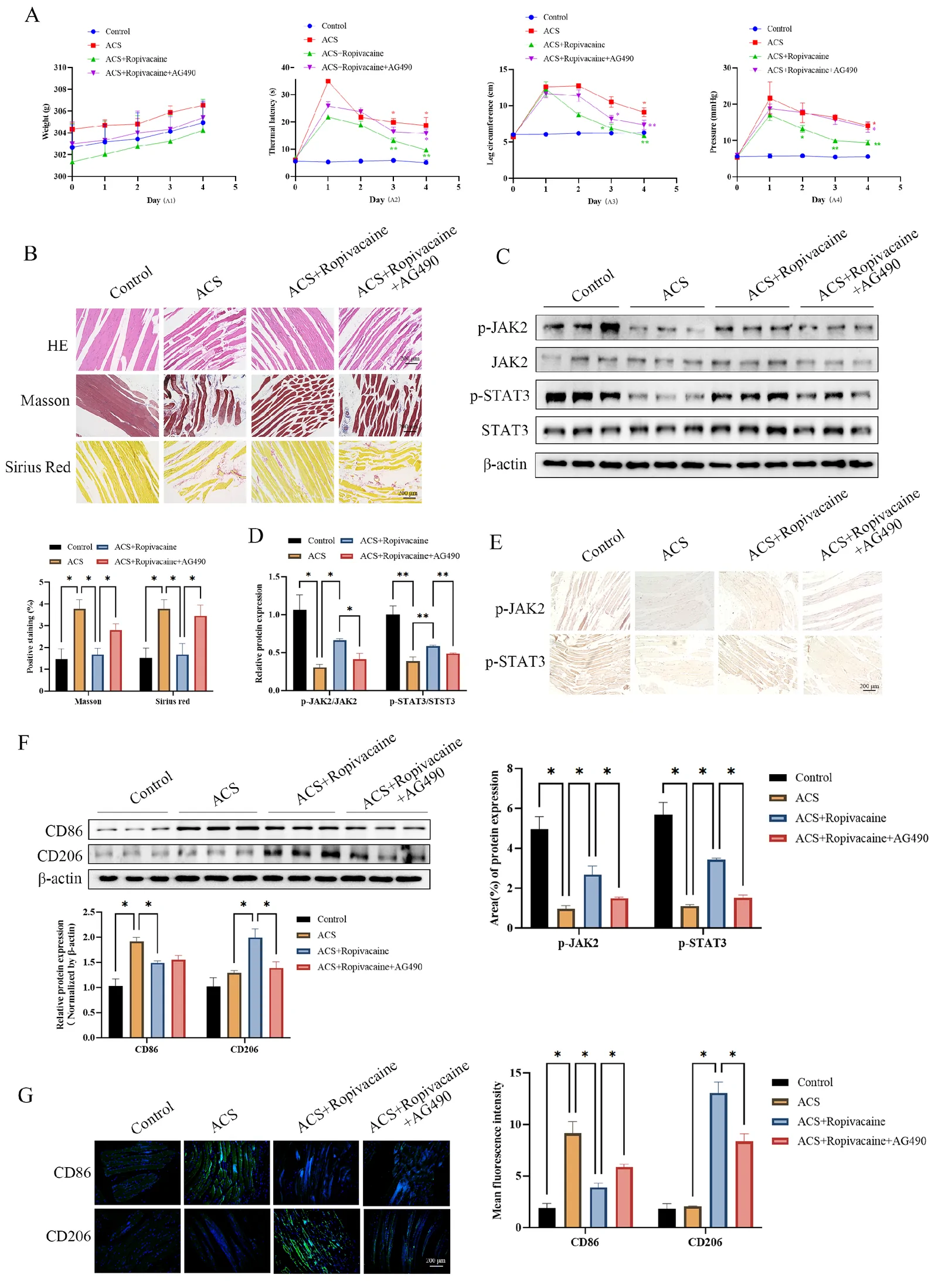
JAK-STAT signaling mediates the protective effects of peripheral nerve block with ropivacaine in acute compartment syndrome in vivo. Rats were subjected to ACS and treated with combined sciatic and femoral nerve block (ropivacaine 0.5%) in the absence or presence of the JAK2 inhibitor AG490 (10 mg/kg, intraperitoneal). (**A**) Functional assessments over 5 days, including body weight (A1), thermal withdrawal latency (A2), leg circumference (A3), and intracompartmental pressure (A4) (n = 6 per group, biological replicates). (**B**) Representative histological staining (HE, Masson’s trichrome, Sirius Red) of muscle tissue sections at day 5. Scale bar = 200 μm. (**C**) Western blot analysis of p-JAK2, JAK2, p-STAT3, STAT3, and β-actin in muscle tissue (n = 3, biological replicates). (**D**) Densitometric quantification of Masson and Sirius Red staining (left panel) and p-JAK2/JAK2 and p-STAT3/STAT3 phosphorylation ratios (right panel) (n = 3, biological replicates). (**E**) Representative IHC images of p-JAK2 and p-STAT3 in muscle tissue sections. Scale bar = 200 μm. The lower panel (**E**): semiquantitative analysis of IHC staining area. (**F**) Western blot analysis of macrophage polarization markers CD86 and CD206 in muscle tissue, with corresponding densitometric quantification (n = 3, biological replicates). (**G**) Representative immunofluorescence images and quantification of CD86 and CD206 in muscle tissue cryosections. Scale bar = 200 μm (n = 6, biological replicates). Error bars represent standard deviation (SD). * *p* < 0.05, ** *p* < 0.01.

Functional outcomes were monitored over the 5-day post-injury period (Figure 5A). Ropivacaine PNB significantly improved all measured functional parameters compared with the untreated ACS group, consistent with the results presented in Figure 1. Ropivacaine-treated rats maintained superior body weight, demonstrated markedly reduced thermal latency responses, exhibited smaller leg circumference measurements, and displayed lower intracompartmental pressures throughout the observation period. However, systemic administration of AG490 completely reversed these ropivacaine-mediated improvements. Rats receiving both ropivacaine PNB and AG490 exhibited body weight trajectories, thermal latency times, leg circumference values, and compartment pressures that were statistically indistinguishable from those of the untreated ACS group and significantly worse than those of the ropivacaine PNB group. These results demonstrate that intact JAK2 signaling is required for the full therapeutic benefit of PNB in ACS.

The functional outcomes were confirmed by histological examination of muscle tissue at the conclusion of the experiment (Figure 5B). HE staining showed that ropivacaine PNB largely maintained muscle fiber structure and decreased inflammation, unlike the severe damage in the ACS group. However, adding AG490 negated this benefit, resulting in disrupted fibers, more necrosis, and inflammation similar to the untreated ACS group. Quantitative assessment of fibrotic deposition by Masson trichrome and Sirius Red staining revealed that ropivacaine PNB significantly reduced collagen deposition and fibrotic remodeling in ACS-injured muscle (Figure 5B,D). AG490 completely blocked this antifibrotic effect, with Masson-positive and Sirius Red-positive staining areas in the PNB plus AG490 group returning to levels statistically comparable to those of the ACS group and significantly higher than those of the ropivacaine PNB group.

To confirm that AG490 effectively blocked JAK-STAT pathway activation in vivo, Western blot analysis of muscle tissue lysates was performed (Figure 5C,D). Ropivacaine PNB significantly increased the phosphorylation of both JAK2 and STAT3 in ACS-injured muscle tissue, as evidenced by elevated p-JAK2/JAK2 and p-STAT3/STAT3 ratios compared with the untreated ACS group. Administration of AG490 completely prevented this PNB-induced activation, with phosphorylation ratios returning to baseline ACS levels. Quantification of p-JAK2/JAK2 and p-STAT3/STAT3 ratios confirmed statistically significant differences between the ropivacaine PNB group and both the ACS and PNB plus AG490 groups. These biochemical findings were corroborated by immunohistochemical staining of muscle tissue sections for JAK-STAT pathway components (Figure 5E). Tissue sections from the ropivacaine PNB group showed strong brown staining for activated p-JAK2 and p-STAT3, indicating increased signaling pathway activation. In contrast, rats co-treated with AG490 had much less staining, similar to the untreated ACS group, suggesting AG490 specifically inhibited kinase activity without affecting total JAK2 and STAT3 protein levels.

The in vivo requirement for JAK-STAT signaling in PNB-mediated macrophage polarization was next examined by Western blot analysis of macrophage phenotype markers in muscle tissue (Figure 5F). Consistent with the above findings, ropivacaine-based PNB significantly increased CD206 and decreased CD86 levels in ACS-injured muscle. This beneficial polarization shift was fully reversed by AG490 (JAK2 inhibitor): compared with the PNB-only group, the PNB + AG490 group showed significantly lower CD206 and higher CD86 expression, with marker levels close to those of the untreated ACS group. Immunofluorescence staining of muscle cryosections validated these Western blot results (Figure 5G). Robust CD206-positive signals and weak CD86 staining were observed in the PNB-treated group, whereas AG490 co-treatment abolished this M2-biased profile. Quantitative immunofluorescence analysis confirmed that JAK2 inhibition blocked PNB-induced M2 macrophage polarization in vivo.

## 4. Discussion

This study reveals two interconnected layers of novel findings regarding ropivacaine-based peripheral nerve block in experimental ACS. First, at the translational therapeutic level, our work provides pre-clinical proof-of-concept that ropivacaine PNB, particularly combined sciatic-femoral nerve block, is not merely an analgesic intervention, but confers direct tissue-protective and anti-inflammatory effects to ameliorate ACS-triggered skeletal muscle injury. To our knowledge, no prior pre-clinical work has reported disease-modifying efficacy of PNB for ACS; regional anesthesia was previously only considered for pain management in ACS patients. Second, at the molecular mechanistic level, using transcriptomic screening together with in vitro and in vivo AG490 pharmacological rescue assays, we pinpoint that the JAK2-STAT3 signaling axis serves as the key downstream mediator by which ropivacaine drives reparative M2 macrophage polarization in an ACS-relevant inflammatory milieu.

In our rat ACS model, ropivacaine PNB markedly improved functional outcomes, preserved muscle histological architecture, mitigated fibrosis, and suppressed local and systemic inflammatory responses. These discoveries hold particular importance due to the significant constraints of existing ACS treatment strategies. Timely surgical fasciotomy can be life- and limb-saving but often results in major complications, including large wounds requiring reconstruction, infection rates exceeding 10%, and long-term issues like chronic venous insufficiency, sensory deficits, and functional limitations [17]. The procedure of fasciotomy does not deal with the inflammation responsible for further tissue harm after ischemia. Differences in outcomes were observed across the three PNB regimens, implying PNB may serve as a valuable therapy beyond just mechanical decompression. Combined sciatic-femoral nerve block showed greater effectiveness than single-nerve block in our experimental setting. Future work is required to determine whether complete neural interruption is required to achieve optimal therapeutic effects for ACS. Ropivacaine’s safety profile, marked by lower cardiotoxicity and central nervous system toxicity than other long-lasting local anesthetics, enhances its clinical viability in this setting [14].

The demonstration that ropivacaine promotes the polarization of macrophages toward the anti-inflammatory M2 phenotype represents a significant extension of prior work and establishes a novel mechanistic basis for its tissue-protective effects in ACS. It is critical to draw a clear distinction between in vitro and in vivo evidence. In cell-culture experiments, we applied ropivacaine directly to LPS/IFN-γ-stimulated RAW264.7 macrophages, demonstrating that ropivacaine is intrinsically capable of directly driving M2 polarization, suppressing pro-inflammatory cytokine secretion, inhibiting macrophage migration, and activating JAK2-STAT3 signaling without contributions from neurons, pain or changes in tissue perfusion. However, these in vitro findings cannot be simply extrapolated to the in vivo setting. In our animal ACS model, ropivacaine was injected around the sciatic and femoral nerves (perineural space), rather than being delivered directly into the injured muscle compartment. After perineural administration, ropivacaine may diffuse locally or enter systemic circulation to reach compartmental macrophages; nevertheless, PNB itself can trigger multiple indirect biological changes that are capable of modulating macrophage polarization independently of direct drug action. These indirect factors include: (1) blockade of afferent nerve signaling and attenuation of pain-driven neuroinflammatory pathways; (2) possible shifts in regional limb microvascular perfusion; (3) reduced secondary tissue injury caused by pain-associated stress responses. Consequently, the observed in vivo M2 macrophage shift and tissue protection in PNB-treated ACS rats likely represent a composite phenotype, potentially arising from a combination of direct ropivacaine immunomodulation and indirect effects mediated by neural blockade. Our current experimental design cannot quantitatively disentangle their relative contributions in vivo.

Notably, the previously reported pro-regenerative effect of ropivacaine on macrophage phenotype was observed in sciatic nerve crush injury and was mediated by Nav1.8-dependent signaling [15]. However, ACS represents a unique pathological context dominated by skeletal muscle ischemia–reperfusion injury rather than primary nerve trauma. Our current study identifies JAK2-STAT3 as a previously unrecognized downstream cascade for ropivacaine-driven macrophage M2 polarization in this ACS-relevant setting, which is independent of the Nav1.8 pathway reported for pure peripheral nerve injury. Our findings are consistent with the recent study by Cui et al. [15], who reported that ropivacaine facilitated nerve regeneration after sciatic nerve injury by promoting an M1-to-M2 phenotypic switch in macrophages through modulation of Nav1.8-mediated signaling [15,18]. The dose–response experiments revealed that ropivacaine at 5 μg/mL produced the most consistent and potent promotion of M2 polarization markers, with higher concentrations (10 μg/mL) showing somewhat attenuated effects. This bell-shaped dose–response relationship is characteristic of many immunomodulatory agents and may reflect the complex concentration-dependent effects of local anesthetics on cellular signaling [14]. In our gradient experiments, 10 μg/mL ropivacaine showed weaker M2-promoting activity compared with 5 μg/mL. Similar non-monotonic dose-responses have been previously reported for local anesthetics on macrophage function [19]. Potential contributing factors may include concentration-dependent off-target cellular effects or subtle cytotoxic stress at higher drug concentrations, although our CCK-8 assay did not detect overt cell death at 10 μg/mL. The exact molecular basis for this attenuation at higher concentrations requires further dedicated experimentation. We also demonstrated that ropivacaine significantly inhibits macrophage migration as assessed by both wound-healing and Transwell assays. This functional consequence of M2 polarization has important implications for the resolution of inflammation in ACS. With a high capacity for movement, M1-polarized macrophages can swiftly penetrate injured areas and consume cellular debris [8]. Nonetheless, the continuous buildup of M1 sustains the inflammatory cycle and leads to collateral tissue damage [7,20]. Shifting to the M2 phenotype reduces migration and inflammatory cell recruitment, promoting tissue repair. In ACS, where limited space and high pressure impede perfusion, minimizing macrophage infiltration and promoting an anti-inflammatory phenotype provide dual tissue protection.

RNA sequencing reveals the JAK-STAT pathway as highly enriched, supporting its role in ropivacaine-induced M2 polarization, consistent with its known function in cytokine-mediated immune regulation [21]. Known for its significant role, the JAK2-STAT3 pathway regulates macrophage polarization when reacting to various cytokine signals [21,22]. JAK2 autophosphorylates after binding to the receptor, which phosphorylates STAT3 at tyrosine 705, resulting in STAT3 homodimerization, nuclear translocation, and the activation of transcription for M2-associated genes like Mrc1 (CD206), Arg1, and IL-10 [21,22]. The study demonstrates that ropivacaine increases p-JAK2/JAK2 and p-STAT3/STAT3 ratios and enhances nuclear localization of these proteins. AG490 completely reversed these effects, indicating JAK-STAT activation is crucial for ropivacaine’s role in M2 polarization and its immunomodulatory effects. The AG490 dose used (10 μM in vitro, 10 mg/kg in vivo) aligns with established protocols that effectively inhibit JAK2-STAT3 signaling in various experiments [23]. Our research identifies a commonly used local anesthetic as a new activator of the JAK2-STAT3 pathway in macrophages and demonstrates its role in tissue protection during ACS. Collectively, these transcriptomic and pharmacological rescue results link our two tiers of novelty together: in vitro, ropivacaine can directly trigger macrophage JAK2-STAT3 activation to drive M2 polarization. In vivo, our AG490 rescue experiments demonstrate that intact JAK2-STAT3 signaling is required for the full beneficial effects of ropivacaine-PNB in ACS. Importantly, this in vivo rescue result confirms that JAK2-STAT3 pathway activity is necessary for the protective outcome, but it still cannot distinguish whether JAK2-STAT3 is activated within macrophages via direct ropivacaine exposure or indirectly via PNB-triggered secondary tissue and immune alterations. When JAK2 is pharmacologically inhibited by AG490, all PNB-derived benefits, including M2 polarization, inflammation suppression, functional recovery and muscle preservation, are fully abrogated.

This study opted for ropivacaine because its pharmacological profile is superior to that of other local anesthetics. Compared with bupivacaine, it is less lipophilic, resulting in a more selective sensory blockade, reduced risk of CNS and cardiovascular toxicity, and a wider therapeutic index [14]. These safety benefits are crucial for ACS patients, particularly those hemodynamically unstable from polytrauma, as motor blockade can impede monitoring for compartment syndrome. It is essential to investigate whether bupivacaine or other amide local anesthetics have similar immunomodulatory effects through JAK-STAT activation. Interestingly, the extended-release liposomal form of bupivacaine did not offer any pain relief advantage over ropivacaine for multimodal periarticular injection in total knee replacement surgery [24], underscoring the practical value of ropivacaine-based regional analgesia. The mechanism identified in this study differs significantly from traditional perioperative anti-inflammatory agents. The main action of NSAIDs is to block cyclooxygenase (COX) enzymes, resulting in reduced synthesis of prostaglandins like PGE2 [25]. Although prostaglandins have the ability to influence macrophage polarization via cAMP-dependent signaling [26], NSAIDs are not targeted regulators of M1/M2 phenotypic switching, and their net effects on macrophage polarization remain indirect and context-dependent. Ropivacaine encourages M2 polarization to modulate inflammation, potentially preserving immune functions and promoting tissue repair, unlike general immunosuppression [14]. This suggests that PNB with ropivacaine could offer a more integrated approach to managing inflammation in ACS [27]. Additionally, exploring synergistic effects between PNB and other anti-inflammatory treatments is a promising area for future research.

This study has some limitations: it used only one time-point for endpoint analysis (5 days post-injury) and had a short follow-up period. While this was enough to observe the acute inflammatory phase and early tissue repair, it may not have fully captured long-term outcomes such as complete functional recovery, fibrotic remodeling, and potential late complications [28,29]. In ACS, macrophage polarization follows a precise sequence, beginning with M1 dominance and slowly transitioning to M2 [7,8]. The 5-day observation likely captures the start of the M2 transition but may not fully reflect the entire timeline of polarization changes or their long-term impact on tissue structure and function [20,30]. Further research with extended timelines is necessary to comprehend PNB-mediated immunomodulation fully. Second, we cannot fully disentangle direct versus indirect mechanisms underlying macrophage phenotypic changes observed *in vivo*. Our in vitro assays confirm that ropivacaine can directly modulate macrophage polarization and JAK2-STAT3 signaling. However, ropivacaine was administered via perineural injection in the ACS animal model. Peripheral nerve blockade may alter afferent neural signaling, pain levels, local perfusion, and secondary tissue injury; all these factors are known to remodel macrophage behavior independent of direct drug exposure. Therefore, the M2-shift observed in PNB-treated ACS rats may reflect a mixture of direct ropivacaine action on tissue macrophages and indirect immune consequences of neural blockade. Future studies, for example using compartment-directed local drug injection, non-immunomodulatory local-anesthetic controls, or myeloid-specific JAK2 conditional knockout animals, will be required to dissect their relative contributions. Third, in vitro mechanistic experiments rely on LPS/IFN-γ-stimulated RAW264.7 macrophages. This simplified inflammatory system is suitable for exploring the direct effect of ropivacaine on macrophages, but it lacks key pathological features of ACS including ischemia–reperfusion, hypoxia, mechanical compression and endogenous DAMP signaling. Therefore, results obtained from this cell model cannot be completely extrapolated to macrophage behavior inside the ACS-injured compartment. Future experiments using primary macrophages and hypoxia/re-oxygenation injury models will help further verify our conclusions. Fourth, we did not include a procedural control group receiving identical nerve-dissection surgery with perineural injection of equal-volume saline (saline-treated nerve-block group). Therefore, we cannot completely separate the specific pharmacological effects of ropivacaine from potential biological responses triggered by the surgical procedure itself (nerve exposure, local soft-tissue manipulation and perineural injection). This control will be necessary for future studies. Furthermore, animals in the PNB groups received repeated daily perineural injection for five consecutive days. We cannot rule out that repeated needle puncture and local injection procedures may induce mild local inflammation or affect tissue recovery. Without a matched repeated-injection procedural control, we are unable to quantitatively disentangle these procedural effects from the pharmacological effects of ropivacaine. This factor should be considered in future experimental designs.

Additionally, the effective ropivacaine concentration of 5 μg/mL was determined from in vitro macrophage assays. We did not measure actual ropivacaine concentrations inside the injured ACS muscle compartment following perineural injection. Thus, we cannot definitively confirm whether this in vitro concentration is pharmacologically relevant to local drug levels achieved in vivo. Tissue drug quantification will be required to establish a more direct connection between our cell-culture observations and in vivo mechanisms in future studies. While this study highlights JAK-STAT signaling in ropivacaine-induced M2 polarization, some aspects of the pathway remain unclear, including the upstream receptors that trigger JAK2-STAT3 activation. Potential candidates include voltage-gated sodium channels, particularly Nav1.8, cytokine receptors such as the IL-6 or IL-10 receptor complexes, and possibly toll-like receptors [12,18]. The research mainly concentrated on the JAK2-STAT3 pathway, leaving the roles of other JAK-STAT variants (such as JAK1, JAK3, TYK2, and additional STAT proteins) largely unexamined [21,22]. In macrophage polarization, STAT1 and STAT6 are important, and their cross-talk with the JAK2-STAT3 pathway may influence the overall phenotypic response [21]. In addition, it should be emphasized that our study does not exclude potential contributions from other cell types or additional signaling pathways triggered by PNB/ropivacaine. Although our rescue experiments demonstrate that macrophage JAK2-STAT3 is a necessary pathway for the observed protection, we cannot rule out ropivacaine’s direct effects on muscle cells, endothelial cells or neurons, which may also partially contribute to the in vivo outcomes. Most of all, the absence of clinical human data represents a significant limitation for immediate translational application. While the rat model provides valuable proof-of-concept evidence, human macrophage biology differs in important respects from murine systems, including differences in receptor expression, cytokine responses, and polarization marker profiles [31]. The concentration of ropivacaine required for M2 polarization in vitro (5 μg/mL) may not be achievable in the human compartment microenvironment at clinically safe doses, and human macrophages may exhibit different sensitivity to ropivacaine’s immunomodulatory effects. Clinical studies will be essential to validate the efficacy and safety of PNB as an adjunctive therapy for ACS in human patients. Finally, only ropivacaine was tested in our experiments. Consequently, we are unable to conclude whether the observed immunomodulatory effects are ropivacaine-specific or reflect a broader class-wide effect shared by other local anesthetics. Direct comparative studies with multiple local anesthetic compounds are needed to resolve this issue in future work.

## 5. Conclusions

In vitro, ropivacaine directly promotes reparative M2 macrophage polarization via activation of the JAK2-STAT3 cascade. In vivo, ropivacaine-based PNB produces disease-modifying protective effects in ACS beyond analgesia, ameliorating muscle inflammation, necrosis, fibrosis and improving functional recovery. These beneficial outcomes require intact JAK2-STAT3 signaling, but may involve a combination of direct ropivacaine immunomodulation and indirect biological effects of PNB.

## Data Availability

The raw RNA-sequencing data generated in this study have been deposited into the Gene Expression Omnibus (GEO) public repository under accession number [GEO: GSE336799]. All other data generated or analyzed during this study are included in this published article.
